# Supplementary material for: Regulatory sites of CaM-sensitive adenylyl cyclase AC8 revealed by cryo-EM and structural proteomics
Source: EMBO Rep. 2024 Feb 13;25(3):31. doi: 10.1038/s44319-024-00076-y (PMC10933263; doi:10.1038/s44319-024-00076-y)
Supplement: Supplementary file 1 — Appendix [file 44319_2024_76_MOESM1_ESM.pdf]

APPENDIX

Regulatory sites of CaM-sensitive adenylyl cyclase AC8 revealed by cryo-EM and structural proteomics

Authors: Basavraj Khanppnavar[1,2,#], Dina Schuster[1,2,3,#], Pia Lavriha[1], Federico Uliana[4], Merve Özel[1], Ved Mehta[1], Alexander Leitner[3], Paola Picotti[3] and Volodymyr M. Korkhov[1,2]*

Affiliations:

[1] Laboratory of Biomolecular Research, Division of Biology and Chemistry, Paul Scherrer Institute, Villigen, Switzerland

[2] Department of Biology, Institute of Molecular Biology and Biophysics, ETH Zurich, Switzerland

[3] Department of Biology, Institute of Molecular Systems Biology, ETH Zurich, Switzerland

[4] Department of Biology, Institute of Biological Chemistry, ETH Zurich, Switzerland

[#] Contributed equally

* Corresponding author. E-mail: volodymyr.korkhov@psi.ch

Table of content:

Appendix Figure S1 ………………………………………………………….. Page 2

Appendix Figure S2 ………………………………………………………….. Page 3

Appendix Figure S3 ………………………………………………………….. Page 4

Appendix Figure S4 ………………………………………………………….. Page 6

Appendix Figure S5 ………………………………………………………….. Page 8

Appendix Figure S6 ………………………………………………………….. Page 9

Appendix Figure S7 ………………………………………………………….. Page 11

Appendix Figure S8 ………………………………………………………….. Page 12

Appendix Figure S9 ………………………………………………………….. Page 13

Appendix Figure S10 ………………………………………………………….. Page 15

Appendix Figure S11 ………………………………………………………….. Page 16

Appendix Table S1 ………………………………………………………….. Page 17

Appendix Table S2 ………………………………………………………….. Page 19

Appendix Table S3 ………………………………………………………….. Page 20

APPENDIX FIGURES

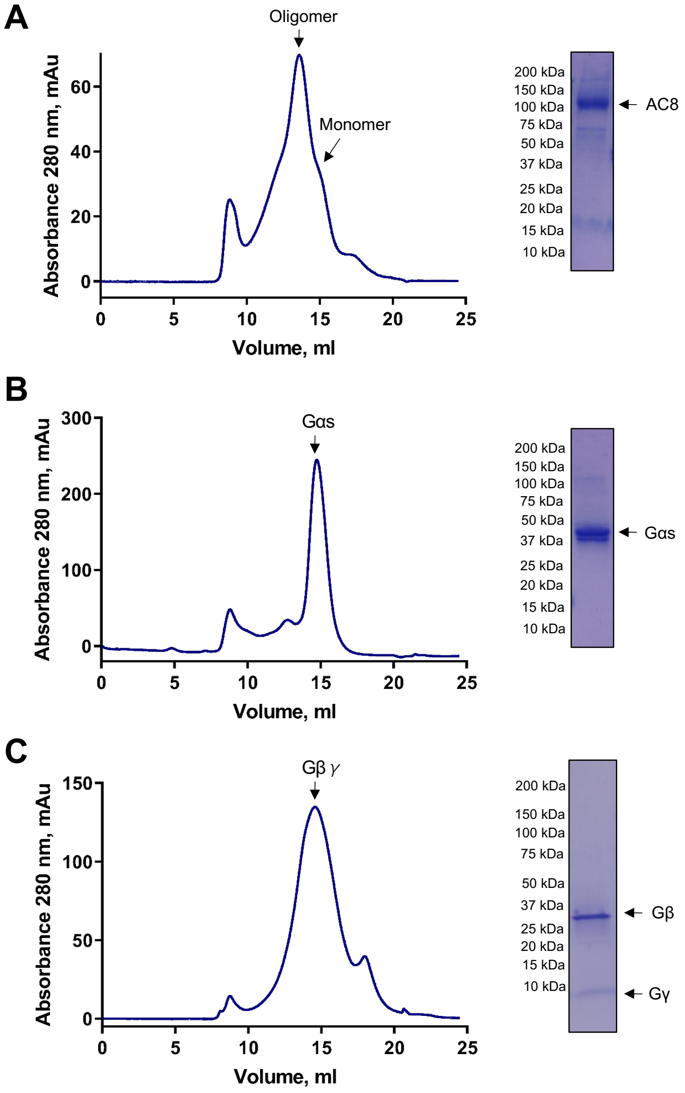

Appendix Figure S1 - Recombinant expression and purification of AC8 and G-protein subunits.

A-C Size exclusion chromatography (SEC) and SDS-PAGE analysis of purified **(A)** bovine AC8, **(B)** G protein Gαs subunit, **(C)** G protein Gβγ subunit. SEC for AC8 and G-proteins was performed using a Superose 6 Increase column.

A

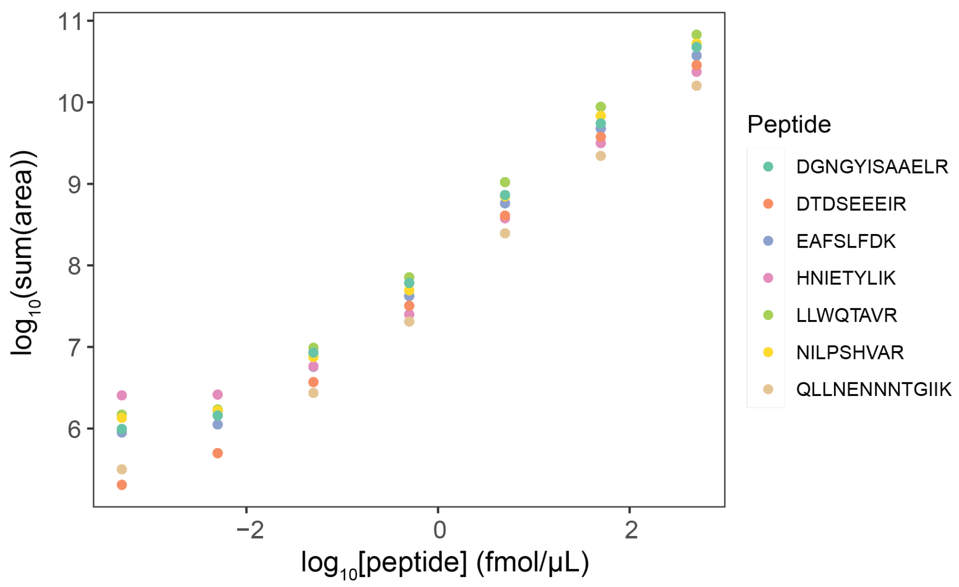

B

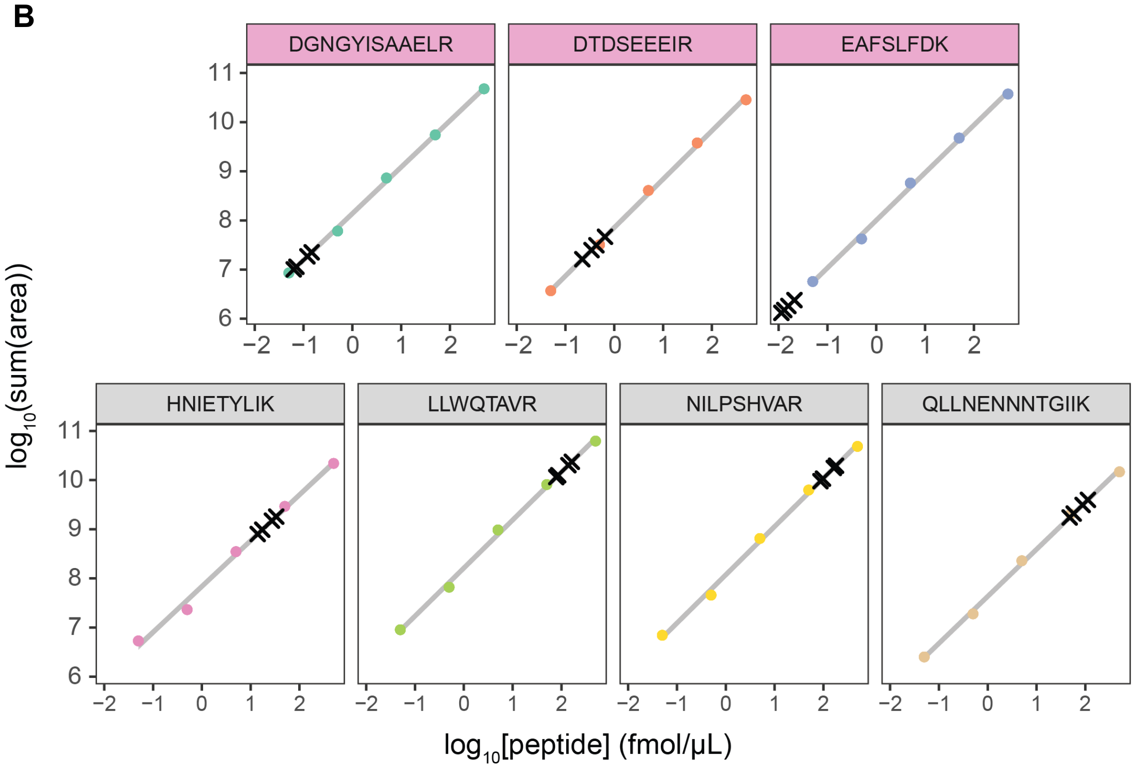

Appendix Figure S2 - PRM calibration curves for AC8 and CaM heavy labeled peptides.

A All calibration points for AC8 (HNIETYLIK, LLWQTAVR, NILPSHVAR, QLLNENNNTGIIK) and CaM (DGNGYISAAELR, DTDSEEIR, EAFSLFDK) heavy labeled peptides.

B Calibration curves for CaM peptides (pink header) and AC8 peptides (grey header). Summed up intensities of the 5 highest ranked fragments of the 0.5 µL and 1 µL injections detected in the samples are shown as black crosses.

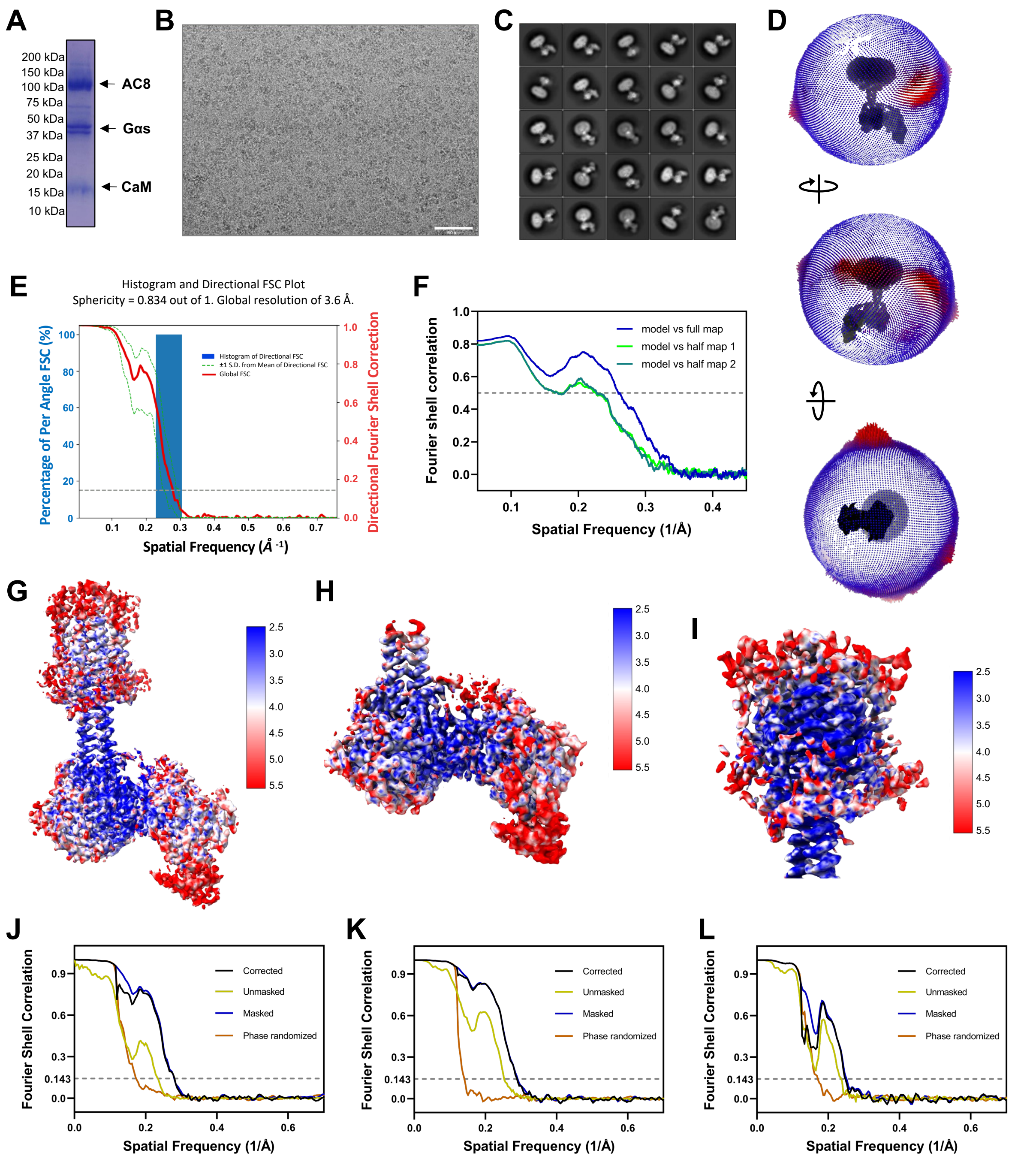

Appendix Figure S3 - Cryo-EM data analysis of AC8-Ca^{2+}/CaM-Gαs-Forskolin-MANT-GTP in detergent.

A SDS-PAGE analysis of AC8-Ca^{2+}/CaM-Gαs (AC8-CaM-Gαs) complex used for cryo-EM analysis.

B A representative micrograph of AC8-CaM-Gαs complex in the presence of 0.5 mM Forskolin, 0.5 mM MANT-GTP, 5 mM $MnCl_2$, 2 mM $MgCl_2$ and1 mM $CaCl_2$.

C Representative 2D classes of AC8-CaM-Gαs.

D Angular distribution histogram of AC8-CaM-Gαs complex dataset.

E 3D FSC and preferred orientation analysis of the dataset with the red line representing the estimated global FSC of 3.6 Å ± 1 SD (green dashed lines). A sphericity of 0.834 indicates absence of severe preferred orientation bias without significant anisotropic map. 3DFSC and preferred orientation analysis was performed using 3DFSC software.

F Model vs. map FSC for the final PHENIX real-space refined model.

G-I Density maps coloured according to local resolution for AC8-CaM-Gαs complex after subtraction of detergent micelle (**G**), after focused refinement of catalytic domain of AC8 and Gαs without TM domain (**H**), after focused refinement of transmembrane domain of AC8 without detergent micelle (**I**).

J-L Fourier shell correlation (FSC) curve of AC8-CaM-Gαs complex after subtraction of detergent micelle (**J**), after focused refinement of catalytic domain of AC8 and Gαs without TM domain (**K**), and after focused refinement of transmembrane domain of AC8 without detergent micelle (**L**).

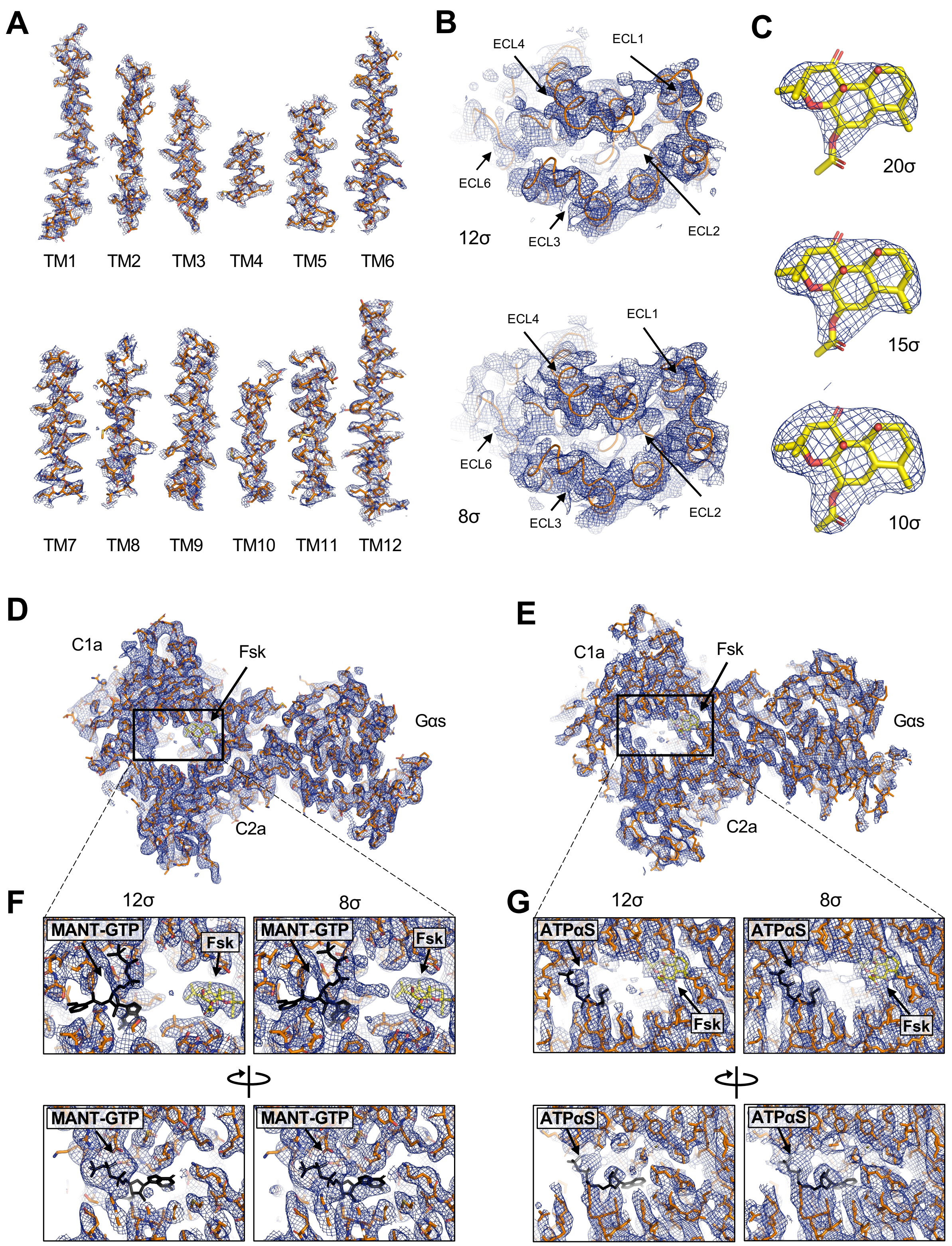

Appendix Figure S4 - Cryo-EM map features of bovine AC8 bound to CaM and Gαs.

A Isolated density map for 12 TM helices of AC8-CaM-Gαs complex in GDN micelles contoured at 10σ threshold level.

B Cryo-EM density features of extracellular loops contoured at 12σ and 8σ levels.

C Cryo-EM density features of forskolin contoured at 20σ, 15σ and 10σ levels.

D-E Cryo-EM density features for the catalytic domain of AC8 bound to Gαs in GDN micelle (panel d) and MSP1ED1 nanodisc (**E**).

F-G Depiction of unresolved density features of MANT-GTP (**F**) and ATPαS (**G**) contoured at 12σ and 8σ levels. MANT-GTP and ATPαS binding poses (shown as black sticks) are, based on structures of chimeric AC5C1a-AC2C2a (PDB: 3C16, 3MAA)

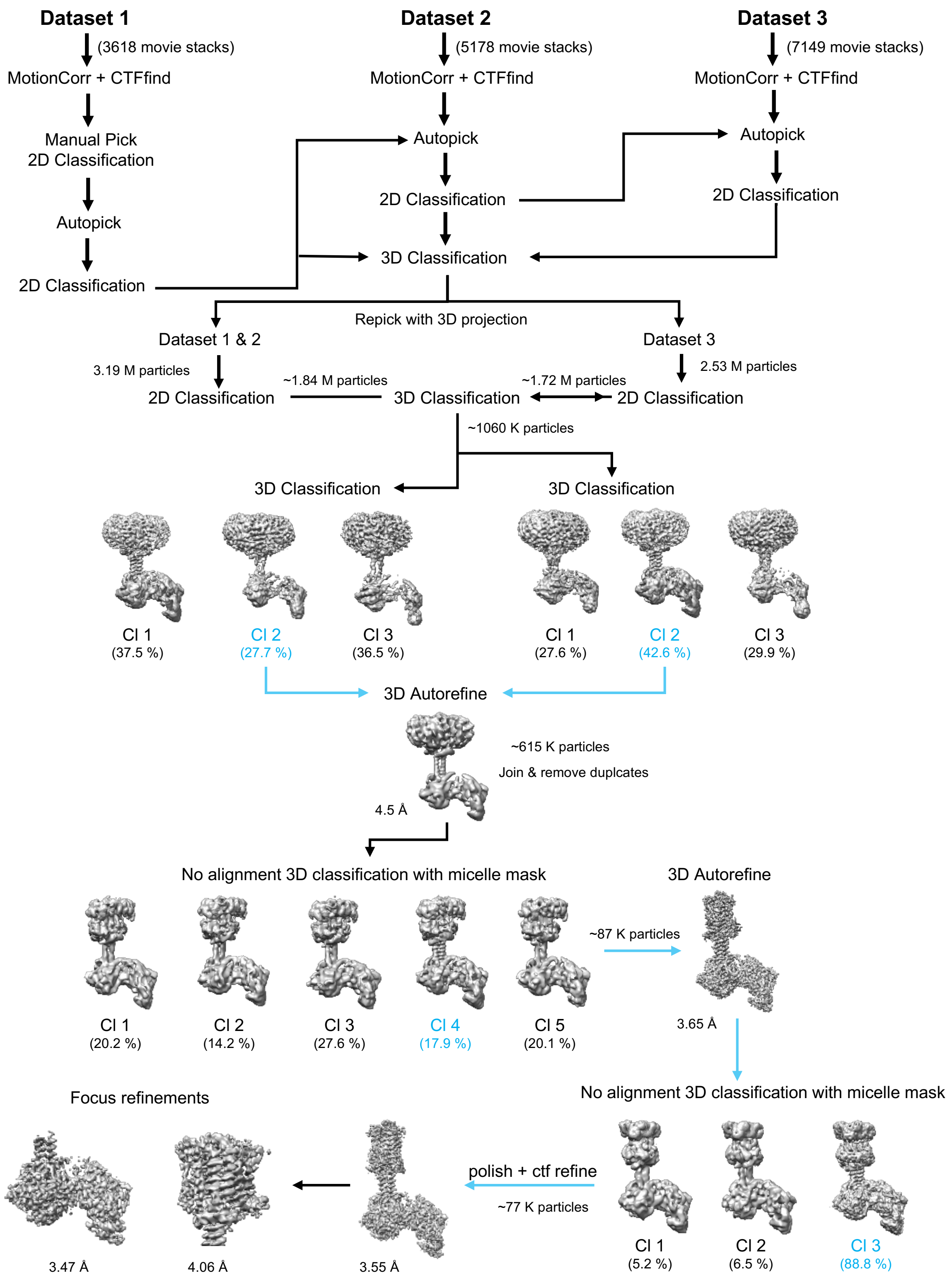

Appendix Figure S5 - Cryo-EM data processing pipeline of AC8-Ca^{2+}/CaM-Gαs-Forskolin-MANT-GTP complex in the detergent micelle.

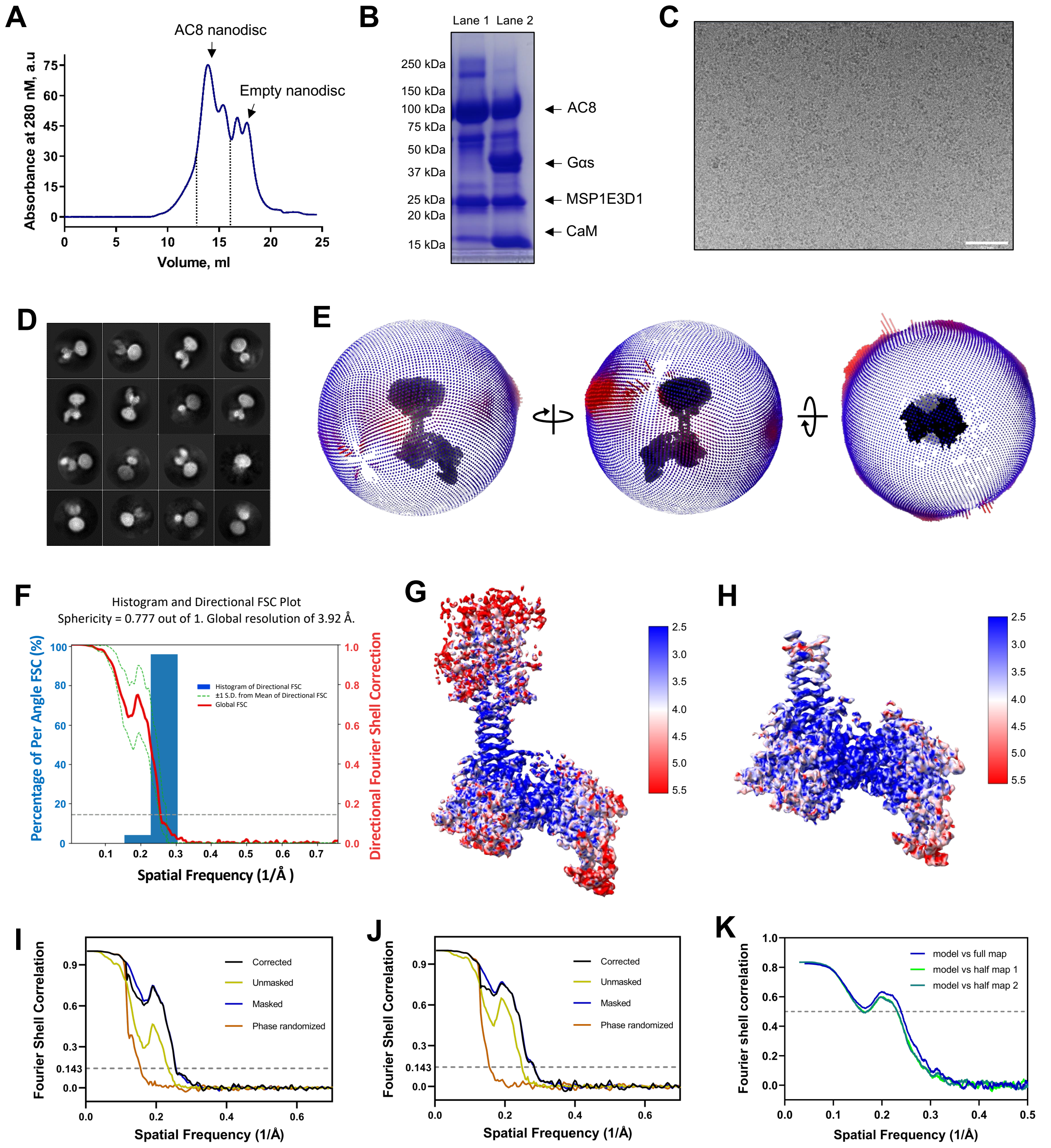

Appendix Figure S6 - Cryo-EM data analysis of AC8-Ca^{2+}/CaM-Gαs-Forskolin-ATPαS complex in lipid nanodisc.

A Size exclusion chromatography (SEC) of AC8 reconstituted in lipid nanodisc with brain polar lipid and MSP1E3D1.

B SDS-PAGE analysis of AC8 nanodisc (lane 1) and cryo-EM sample of AC8-Ca^{2+}/CaM-Gαs complex in lipid nanodisc (lane 2).

C A representative micrograph of AC8-CaM-Gαs complex in the presence of 0.5 mM Forskolin, 1 mM ATPαS, 5 mM $MnCl_2$, 2 mM $MgCl_2$ and 1 mM $CaCl_2$.

D Representative 2D classes of AC8-CaM-Gαs in nanodisc.

E Angular distribution histogram of AC8-CaM-Gαs complex in nanodisc.

F 3D FSC and preferred orientation analysis of the dataset with the red line representing the estimated global FSC of 3.92 Å ± 1 SD (green dashed lines). A sphericity of 0.777 indicates absence of severe preferred orientation bias and without significant anisotropy in the final map. 3DFSC and preferred orientation analysis was performed using 3DFSC software.

G-H Density maps coloured according to local resolution for AC8-CaM-Gαs complex after subtraction of detergent micelle (**G**), and after focused refinement of catalytic domain of AC8 and Gαs without TM domain (**H**).

I-J Fourier shell correlation (FSC) curve of AC8-CaM-Gαs complex after subtraction of detergent micelle (**I**), after focused refinement of catalytic domain of AC8 and Gαs without TM domain (**J**).

K Model vs. map FSC for the final PHENIX real-space refined model.

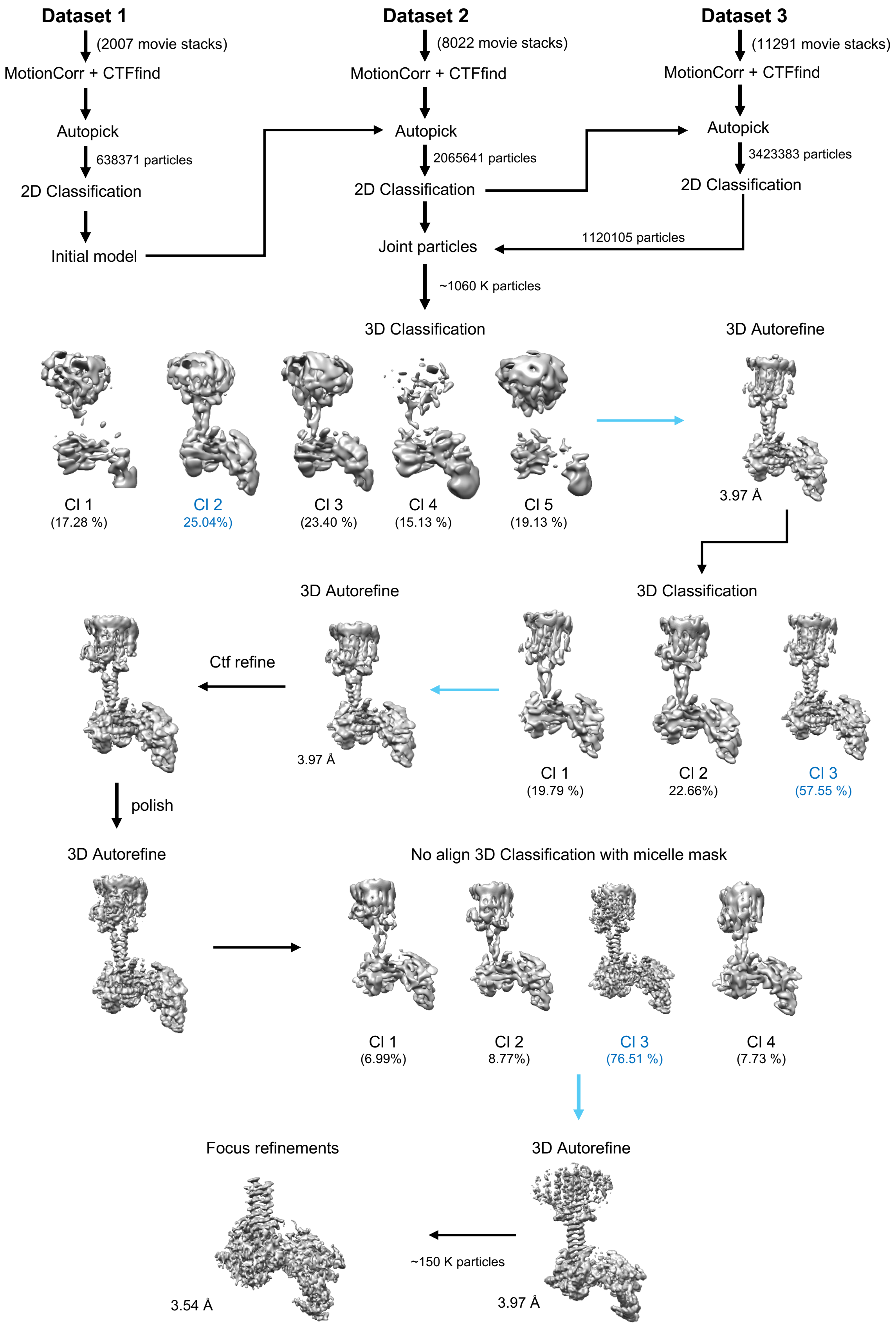

Appendix Figure S7 - Cryo-EM data processing pipeline of AC8-Ca^{2+}/CaM-Gαs-Forskolin-ATPαS complex in lipid nanodisc.

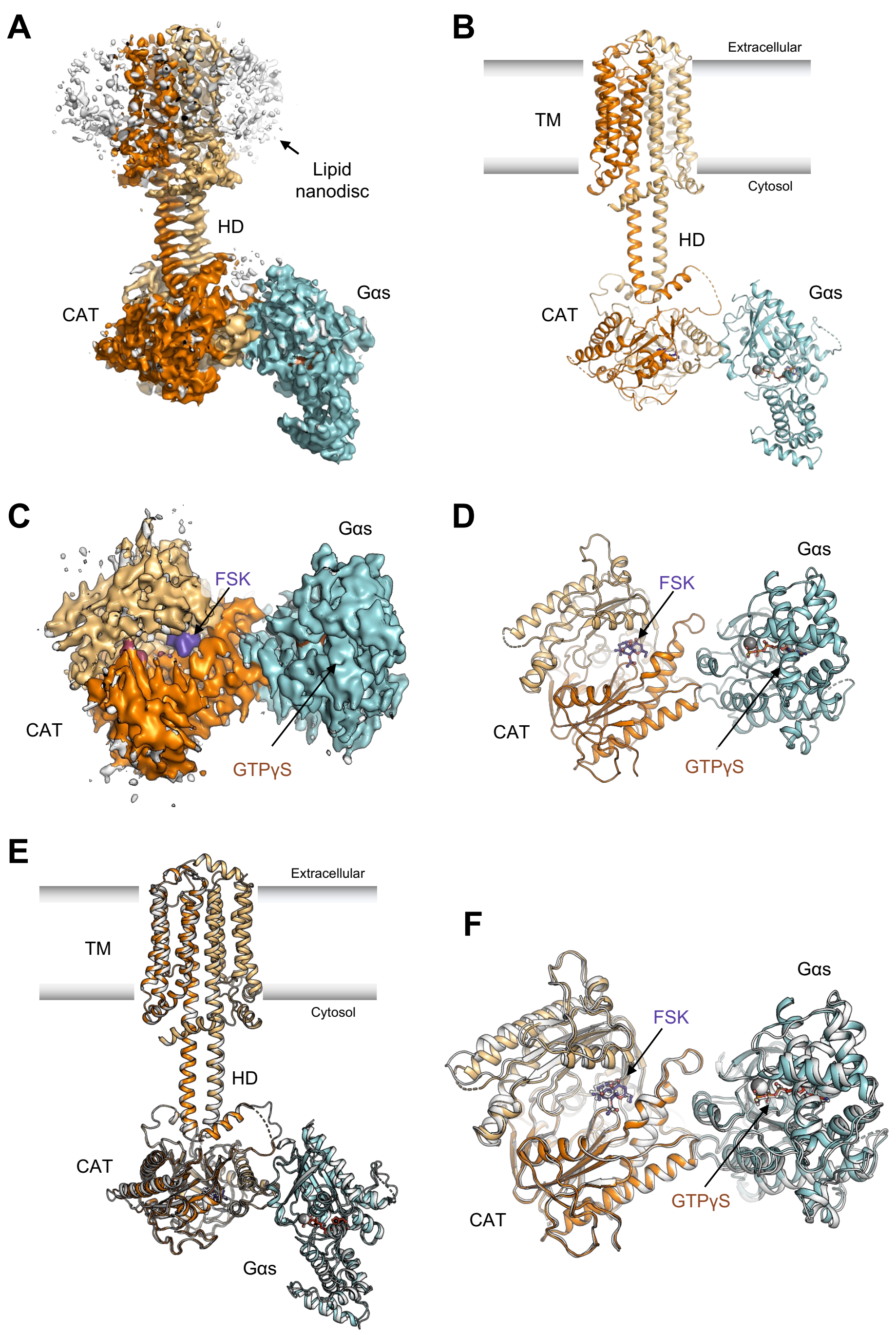

Appendix Figure S8 - Cryo-EM structure of AC8-CaM-Gαs structure in lipid nanodisc.

A-D Cryo-EM map and model of AC8-CaM-Gαs-Forskolin-ATPαS complex reconstituted in lipid nanodisc (MSP1E3D1 and brain polar lipid).

E-F Comparison of the cryo-EM structures of AC8-Gαs complex resolved in detergent (white) and lipid nanodisc (orange).

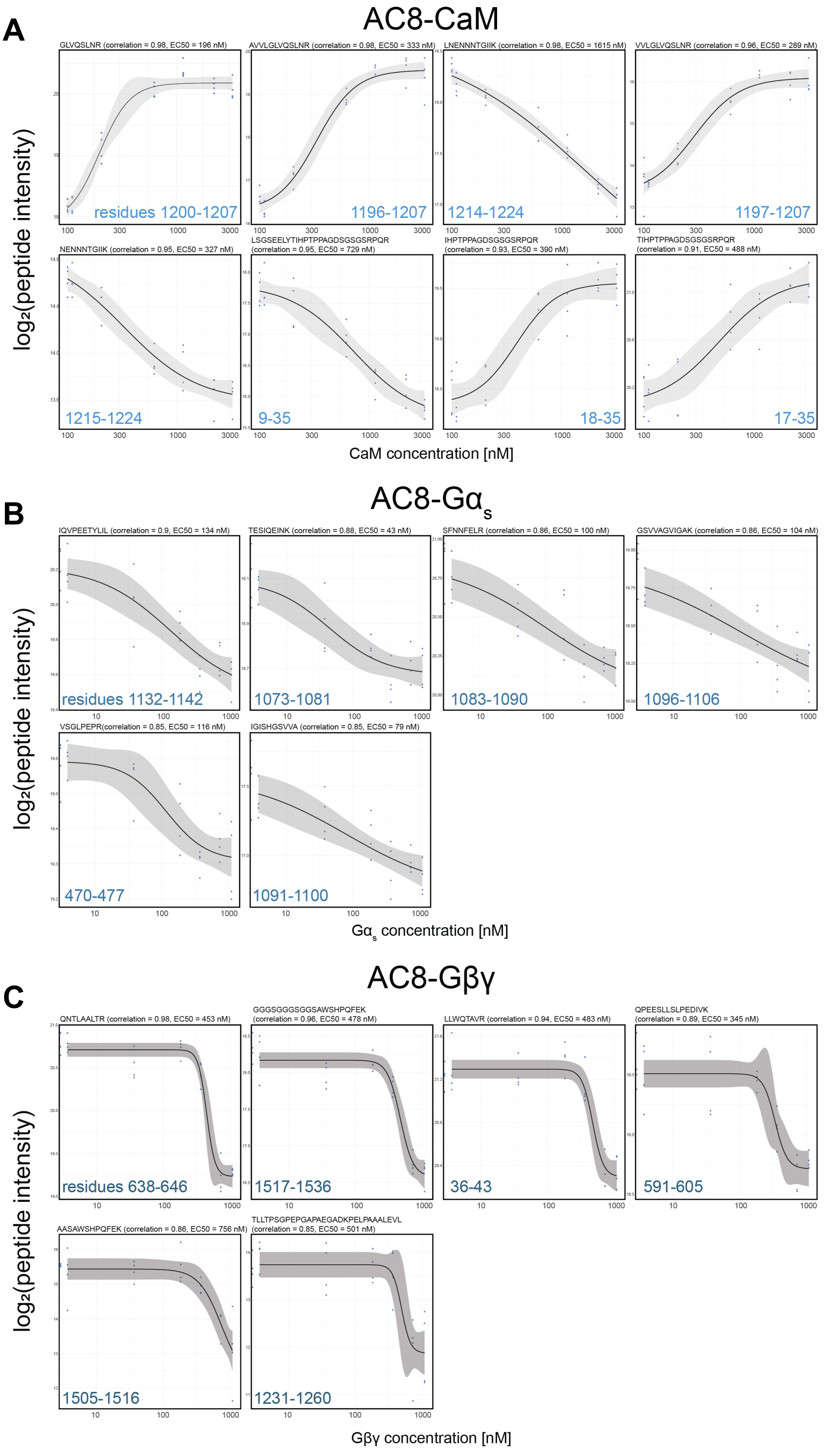

Appendix Figure S9 - Limited proteolysis mass spectrometry (LiP-MS) analysis of AC8 complexes.

A-C LiP-MS curves for all peptides with Pearson r > 0.85 for AC8-CaM (**A**), AC8-Gαs (**B**) and AC8-G$\beta\gamma$ (**C**) titrations. Peptide sequences are shown in the header of each plot, alongside the Pearson r (correlation) and EC_{50} values. The peptide positions within the AC8 sequence are indicated at the bottom of each plot. The measured and log2 transformed peptide intensities for each replicate at each interactor concentration are shown as blue dots. The 95 % model confidence interval is shown as a grey shaded area. Peptides with only one changing condition were excluded.

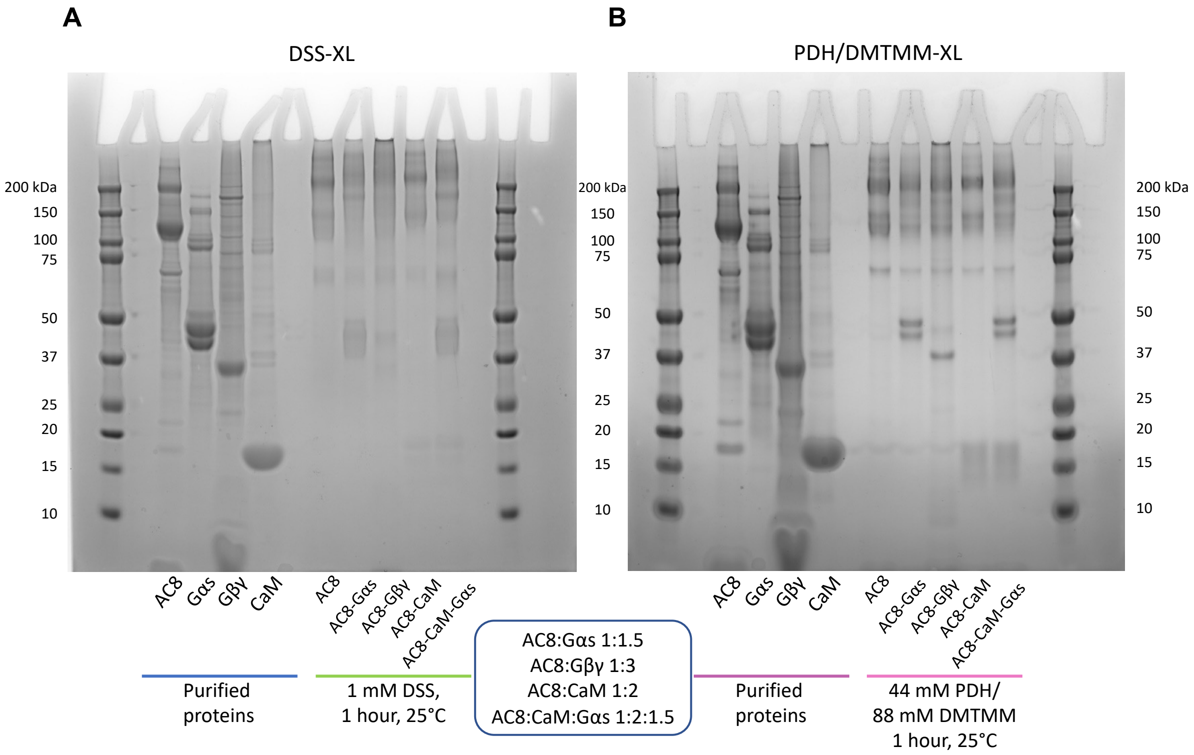

Appendix Figure S10 - SDS-PAGE of purified and crosslinked proteins (DSS and PDH/DMTMM).

A SDS-PAGE of purified and DSS-crosslinked proteins. The migration distances of the respective proteins are indicated with a blue arrow on the left side of the gel.

B SDS-PAGE of purified and PDH/DMTMM-crosslinked proteins**.** Molar ratios of the crosslinked proteins are listed in the blue box in the center. Reaction conditions are listed below.

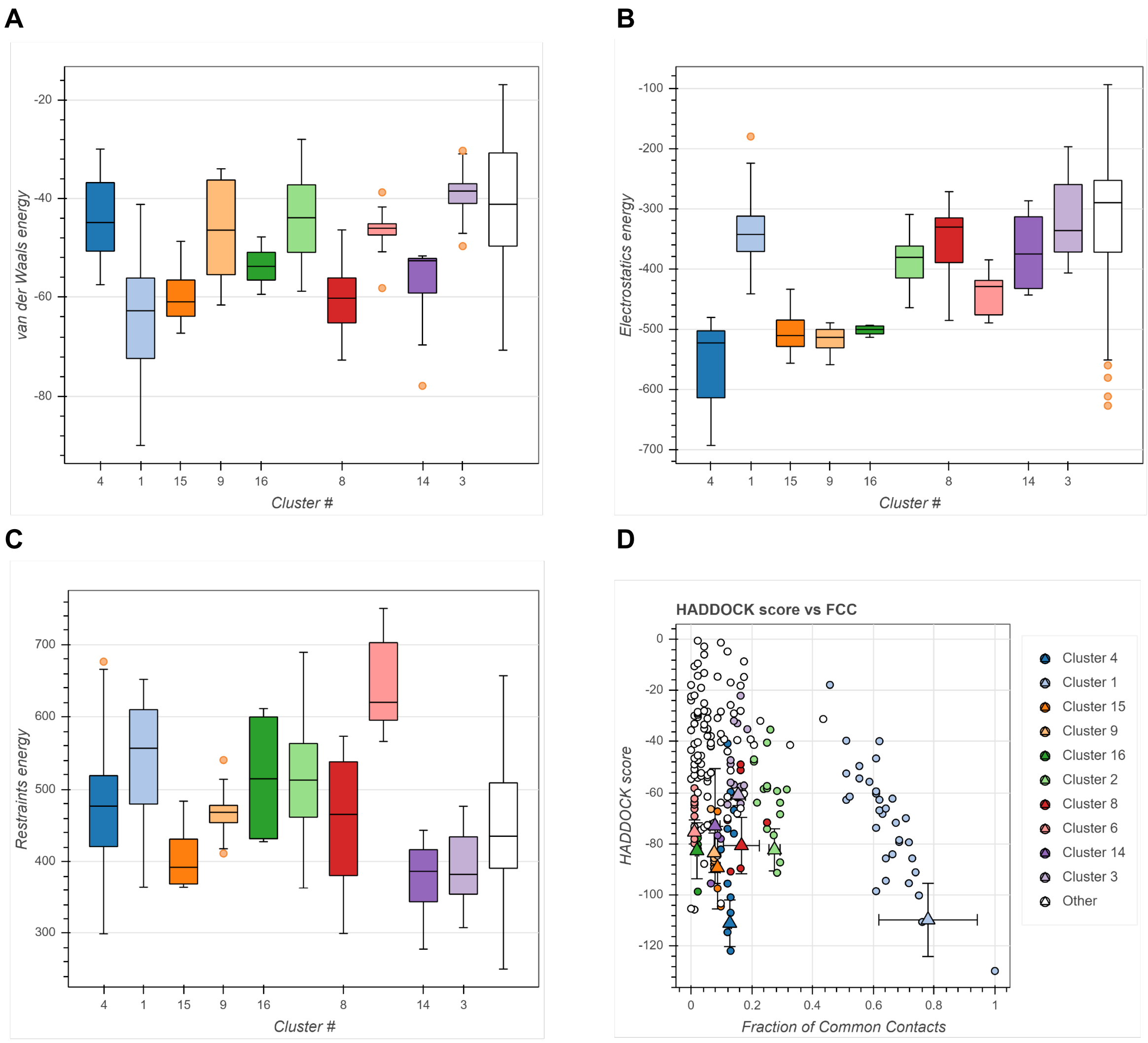

Appendix Figure S11 - HADDOCK quality control plots.

A Van der Waals energy ranges for the different docking clusters generated by HADDOCK. The standard box plots are defined as follows: the box is drawn from the first quartile (Q1) to the third quartile (Q3), with a line indicating the median (Q2); the whiskers indicate the range in the remaining data, including the minimum (Q0) and the maximum values (Q4).

B Electrostatics energy distributions for the different clusters.

C Restraints energies for the generated clusters.

D Dot plot showing the HADDOCK score vs. fraction of common contacts. The plots were generated in HADDOCK 2.4. Each dot represents a model and the colour of the dot indicates the cluster to which it belongs. The cluster averages with standard deviations are shown as coloured triangles with associated error bars. Cluster 1 shows the lowest mean van der Waals energy, the highest fraction of common contacts and a plausible orientation of Gβγ that would facilitate membrane anchoring.

APPENDIX TABLES

Appendix Table S1. PRM method parameters. Acquisition parameters used for the PRM mass spectrometry method. Higher Orbitrap resolution settings and longer injection times were used for the peptides relevant for absolute quantification.

Protein	Peptide	Precursor (m/z)	Precursor Charge (z)	Collision Energies (%)	Orbitrap Resolution	Normalised AGC Target (%)	Maximum Injection Time (ms)	Polarity
(iRT)	ADVTPADFSEWSK (light)	726.8357	2	27	15000	400	22	Positive
(iRT)	VEATFGVDESNAK (light)	683.8279	2	27	15000	400	22	Positive
(iRT)	YILAGVENSK (light)	547.298	2	27	15000	400	22	Positive
(iRT)	DGLDAASYYAPVR (light)	699.3384	2	27	15000	400	22	Positive
(iRT)	GAGSSEPVTGLDAK (light)	644.8226	2	27	15000	400	22	Positive
(iRT)	GTFIIDPAAVIR (light)	636.8692	2	27	15000	400	22	Positive
(iRT)	GTFIIDPGGVIR (light)	622.8535	2	27	15000	400	22	Positive
(iRT)	LFLQFGAQGSPFLK (light)	776.9298	2	27	15000	400	22	Positive
(iRT)	LGGNEQVTR (light)	487.2567	2	27	15000	400	22	Positive
(iRT)	TPVISGGPYEYR (light)	669.8381	2	27	15000	400	22	Positive
(iRT)	TPVITGAPYEYR (light)	683.8537	2	27	15000	400	22	Positive
CaM	EAFSLFDK (light)	478.7398	2	27	60000	400	118	Positive
CaM	EAFSLFDK (heavy)	482.7469	2	27	60000	400	118	Positive
AC8	LLWQTAVR (light)	493.7927	2	27	60000	400	118	Positive
AC8	LLWQTAVR (heavy)	498.7969	2	27	60000	400	118	Positive
AC8	HNIETYLIK (light)	565.8139	2	27	60000	400	118	Positive
AC8	HNIETYLIK (heavy)	569.821	2	27	60000	400	118	Positive
AC8	NILPSHVAR (light)	503.7933	2	27	60000	400	118	Positive
AC8	NILPSHVAR (heavy)	508.7974	2	27	60000	400	118	Positive
AC8	QLLNENNNTGIIK (light)	735.8992	2	27	60000	400	118	Positive

AC8	QLLNENNNTGIIK (heavy)	739.9063	2	27	60000	400	118	Positive
CaM	DTDSEEEIR (light)	547.2358	2	27	60000	400	118	Positive
CaM	DTDSEEEIR (heavy)	552.24	2	27	60000	400	118	Positive
CaM	DGNGYISAAELR (light)	633.3097	2	27	60000	400	118	Positive
CaM	DGNGYISAAELR (heavy)	638.3138	2	27	60000	400	118	Positive

Appendix Table S2. Cryo-EM analysis and statistics

**Data collection**						
	**AC8-CaM-Gαs in GDN micelle**			**AC8-CaM-Gαs in lipid nanodisc**		
	Full	sAC8-Gαs	tmAC8	Full	sAC8-Gαs	tmAC8
Instrument	FEI Titan Krios / Gatan K3 Summit			FEI Titan Krios / Gatan K3 Summit		
Magnification	130000			130000		
Voltage (kV)	300			300		
**Electron Dose (e-/Å2)**						
Data-set 1	60 e-/Å^2			55 e-/Å^2		
Data-set 2	56 e-/Å^2			55 e-/Å^2		
Data-set 3	49 e-/Å^2			56.4 e-/Å^2		
Defocus range (μm)	-0.6 to -3.0			-0.6 to -3.0		
Pixel size (Å)	0.66			0.66		
**Refinement**						
Number of particles	77575			150262		
Map symmetry	C1			C1		
Model resolution at FSC threshold 0.143	3.50 Å	3.38 Å	4.13 Å	3.97 Å	3.54 Å	4.20 Å
Map sharpening B-factor (Å)	-80	-80	-120	-80	-80	-188
Map CC	0.76	-	-		0.65	-
**Model composition**						
Protein residues/ligands	1223/3			767/3		
Bond length (r.m.s.d)	0.010			0.012		
Bond angle (r.m.s.d)	0.630			0.678		
**Validation**						
MolProbity score	1.92			2.14		
Clashscore	11.73			17.89		
Rotamer outlier (%)	0.46			0.59		
**Ramachandran plot**						
Favored (%)	95.21			94.27		
Allowed (%)	4.79			5.73		
Disallowed (%)	0			0		

Appendix Table S3. Conserved and variable residues in AC-Gαs binding interfaces

Protein	Gαs	AC8	AC9	$AC2_{(C1a)}$-$AC5_{(c2a)}$
Interacting residues	K223	-	E1073	-
	Q236	Q1005	-	-
	Q236	-	-	N905
	Q236	-	-	C911
	**N239***	**R1011**	**R1084**	**R913**
	N239	**-**	**E1081**	**-**
	**R280**	**D1019**	**D1092**	**D921**
	R280	E1015	-	E917
	T284	**-**	**-**	M378

* The conserved interacting residue pairs in all three AC isoforms are bold
