# Supplementary figures and images for: Regulatory sites of CaM-sensitive adenylyl cyclase AC8 revealed by cryo-EM and structural proteomics
Source: EMBO Rep. 2024 Feb 13;25(3):31. doi: 10.1038/s44319-024-00076-y (PMC10933263; doi:10.1038/s44319-024-00076-y)

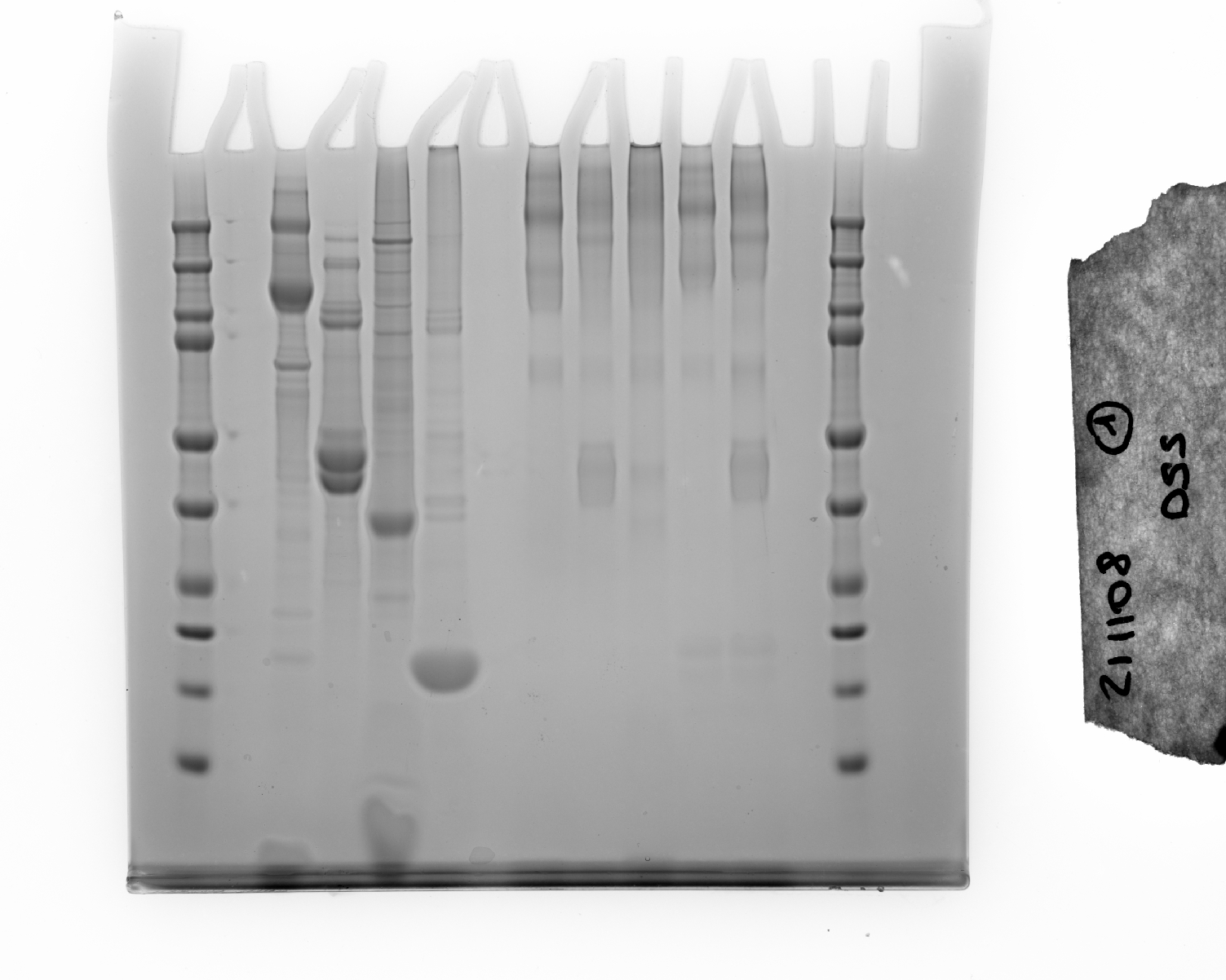

Supplement: Supplementary file 4 — EV and Appendix Figures Source Data [file 44319_2024_76_MOESM4_ESM.zip › Appendix Figure S10/S10A/211108_SD23_XL_DSS(Coomassie Blue).jpg]

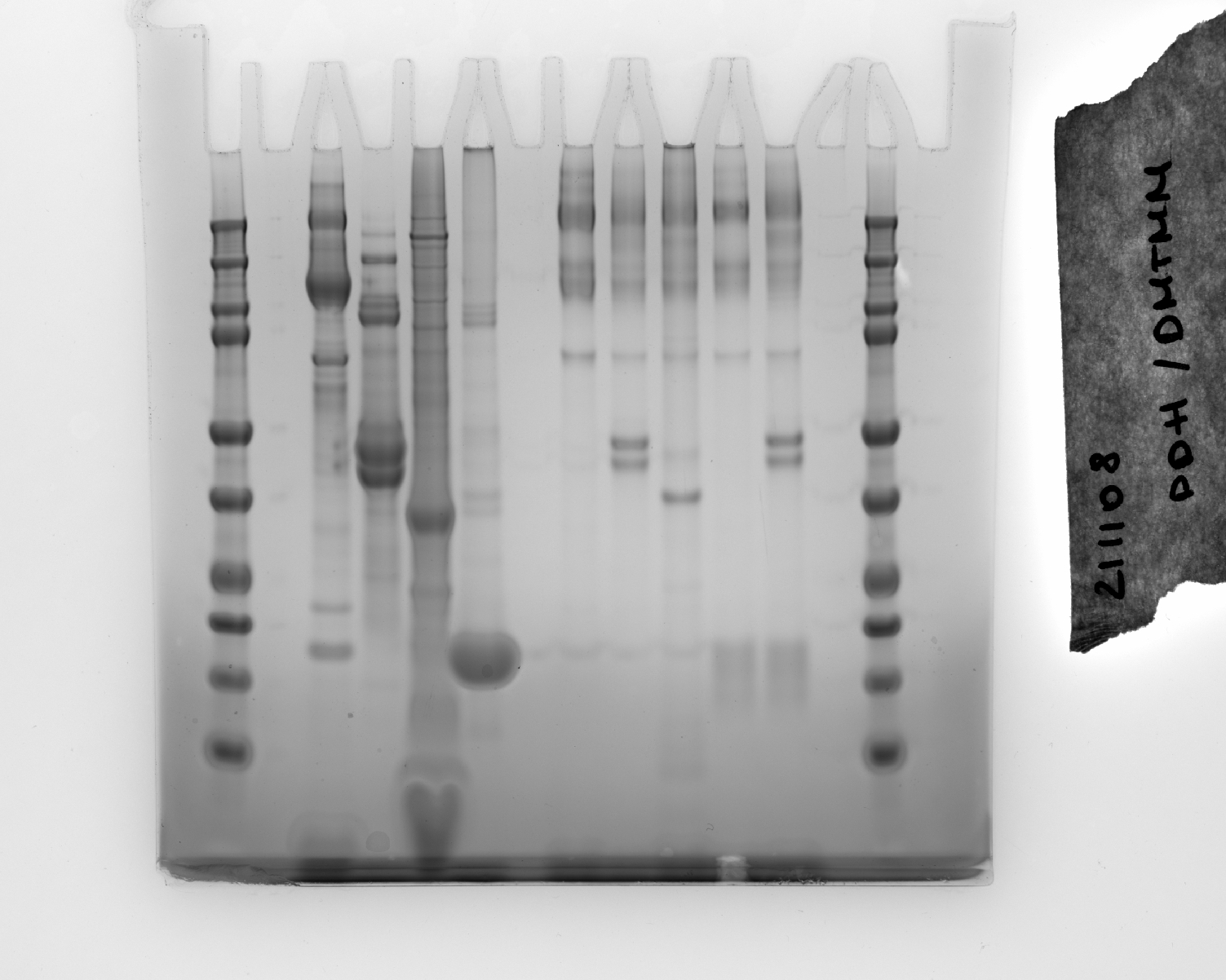

Supplement: Supplementary file 4 — EV and Appendix Figures Source Data [file 44319_2024_76_MOESM4_ESM.zip › Appendix Figure S10/S10B/211108_SD23_XL_PDH(Coomassie Blue).jpg]
