# Supplementary material for: Regulatory sites of CaM-sensitive adenylyl cyclase AC8 revealed by cryo-EM and structural proteomics
Source: EMBO Rep. 2024 Feb 13;25(3):31. doi: 10.1038/s44319-024-00076-y (PMC10933263; doi:10.1038/s44319-024-00076-y)
Supplement: Supplementary file 6 — Expanded View Figures [file 44319_2024_76_MOESM6_ESM.pdf]

Expanded View Figures

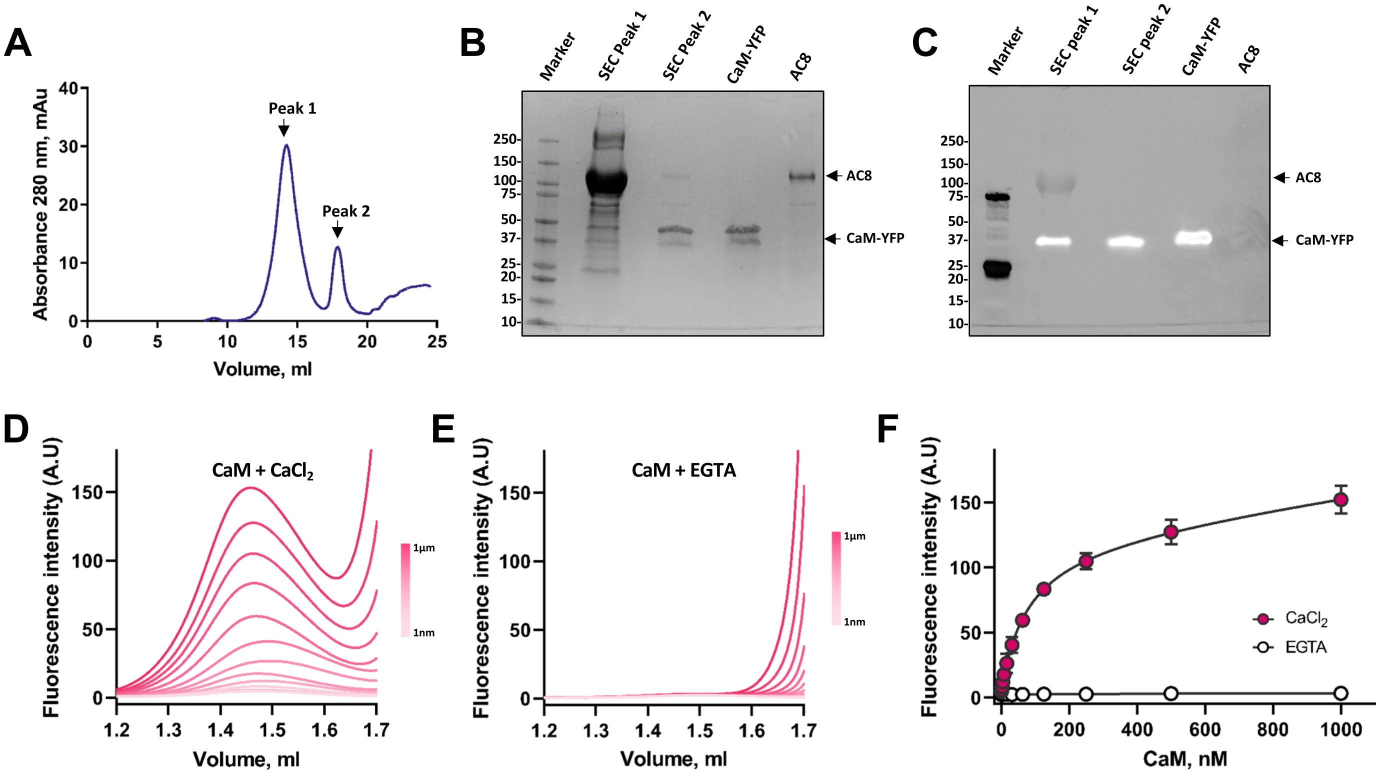

Figure EV1. Purified AC8 binds CaM with high affinity in the presence of Ca^{2+}.

(**A**) The size exclusion chromatography (SEC) profile of the AC8-Ca^{2+}/CaM-YFP complex. (**B**, **C**) SDS-PAGE analysis of AC8-Ca^{2+}/CaM-YFP complex. The complex was prepared by mixing purified recombinant AC8 and CaM-YFP in 1:2 ratio, respectively. The panel (**B**) shows the Coomassie stained SDS-PAGE of AC8-Ca^{2+}/CaM complex after SEC. Panel (**C**) shows overlayed prestained and YFP-fluorescence images of the SDS-PAGE displayed in panel (**B**). (**D**, **E**) Representative FSEC profiles an increase in fluorescence intensity of the AC8 elution peak with increasing concentration of CaM. (**F**) The saturation-binding curves show nanomolar affinity (77.7 ± 10.9 nM, $n = 4$, technical replicates) of AC8 for CaM in presence of calcium and no AC8-CaM interaction in absence of calcium. For the experiments in panel (**F**), the data are shown as mean ± S.E.M. ($n = 4$). Source data are available online for this figure.

 © The Author(s)

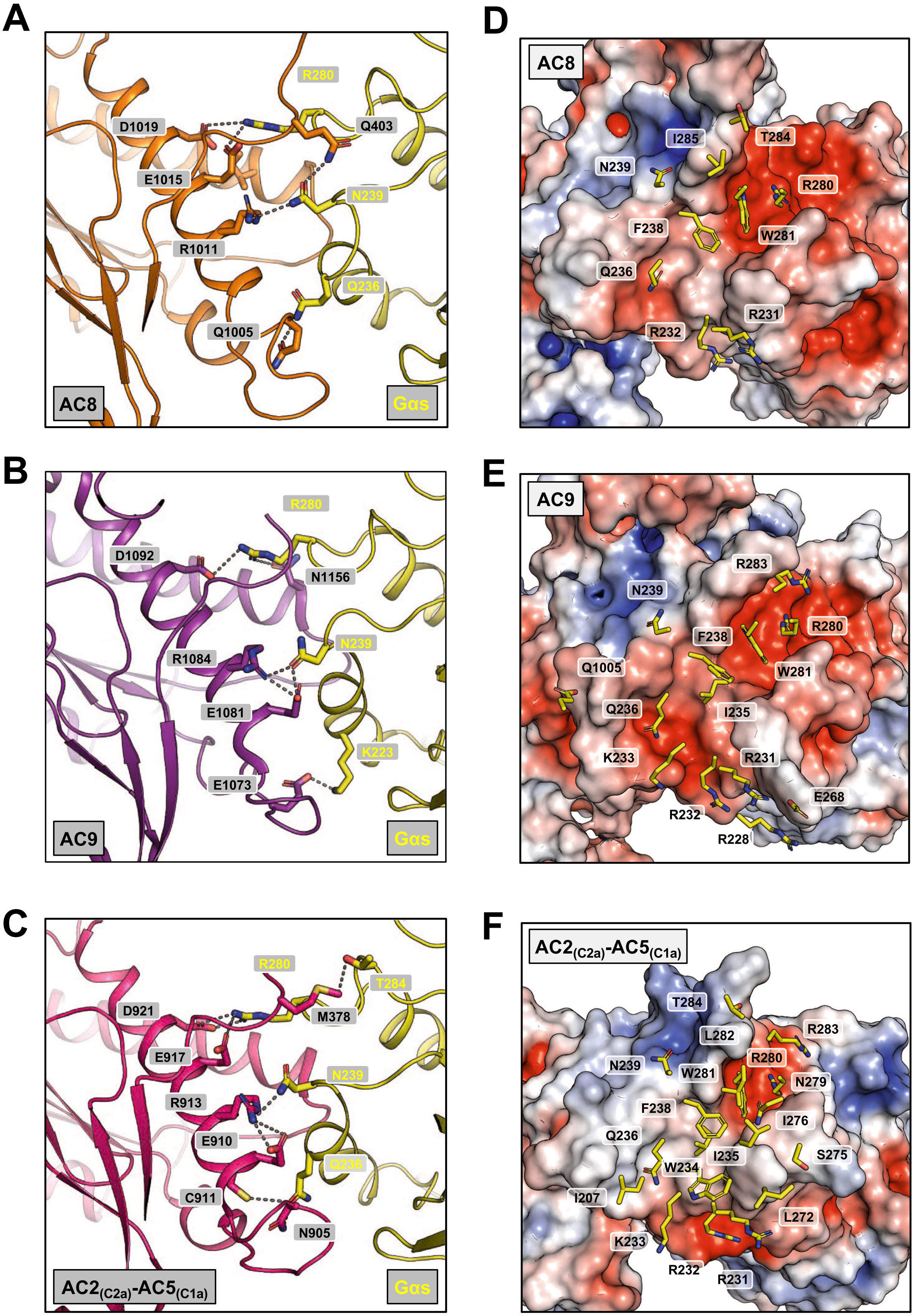
A
R280
D1019
Q403
E1015
N239
R1011
Q236
Q1005
AC8
Gαs
B
R280
D1092
N1156
N239
R1084
E1081
K223
E1073
AC9
Gαs
C
R280
T284
D921
M378
E917
N239
R913
E910
Q236
C911
N905
AC2(C2a)-AC5(C1a)
Gαs
D
AC8
I285
T284
N239
R280
F238
W281
Q236
R231
R232
E
AC9
R283
N239
R280
F238
Q1005
W281
Q236
I235
K233
R231
E268
R232
R228
F
AC2(C2a)-AC5(C1a)
T284
L282
R283
N239
R280
W281
N279
F238
I276
Q236
I235
S275
W234
I207
L272
K233
R232
R231

Figure EV2. Gαs binding interfaces in ACs.

(**A–C**) Depiction of AC-Gαs interfacial residues observed in the cryo-EM structure of AC8-CaM-Gαs (top), AC9-Gαs (middle), and crystal structure of chimeric AC2$_{(C2a)}$-AC5$_{(C1a)}$-Gαs complex (bottom). (**D–F**) Electrostatic surface representation of AC8 (top), AC9 (middle), and chimeric AC2$_{(C2a)}$-AC5$_{(C1a)}$ (bottom) showing interfacial residues of Gαs (yellow colour) within 4 Å of AC.

 © The Author(s)

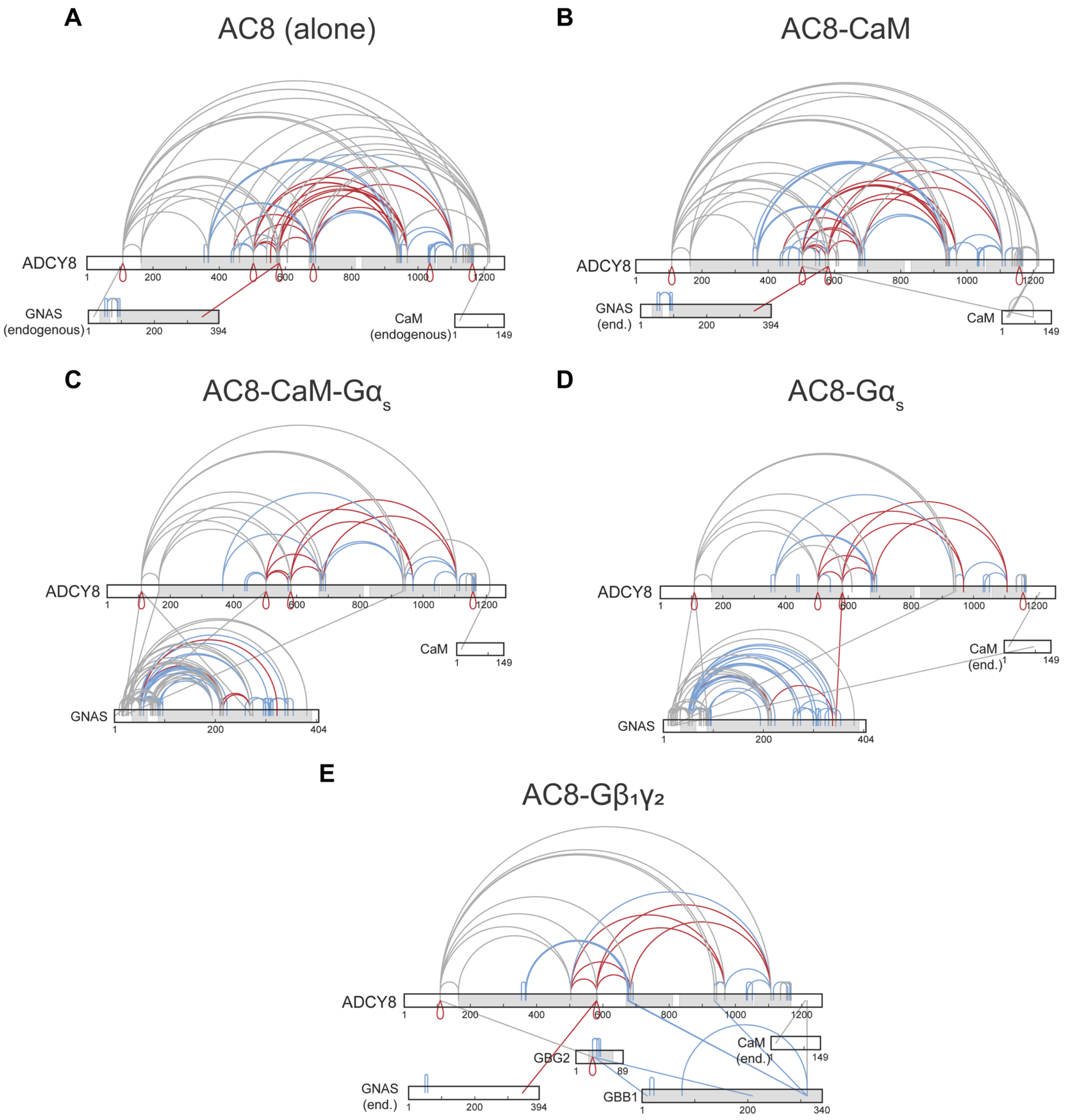

Figure EV3. All detected crosslinks (DSS and PDH/DMTMM).

(**A**) Crosslinked AC8 with copurified Gαs and CaM. Violated crosslinks (>30 Å) are coloured in red, satisfied crosslinks between structured/resolved regions are coloured in blue. Crosslinks that involve flexible regions and regions that are not resolved in the cryo-EM or in the structures used for protein-protein docking are coloured in grey. Homomultimeric links (oligomerisation links) are indicated with red drops. Regions with available structure are highlighted in grey on the respective protein sequence. (**B**) Crosslinked AC8 and CaM with copurified Gαs. (**C**) Crosslinked AC8, CaM and Gαs. (**D**) crosslinked AC8 and Gαs with copurified CaM (**E**) crosslinked AC8 and Gβγ with copurified Gαs and CaM.

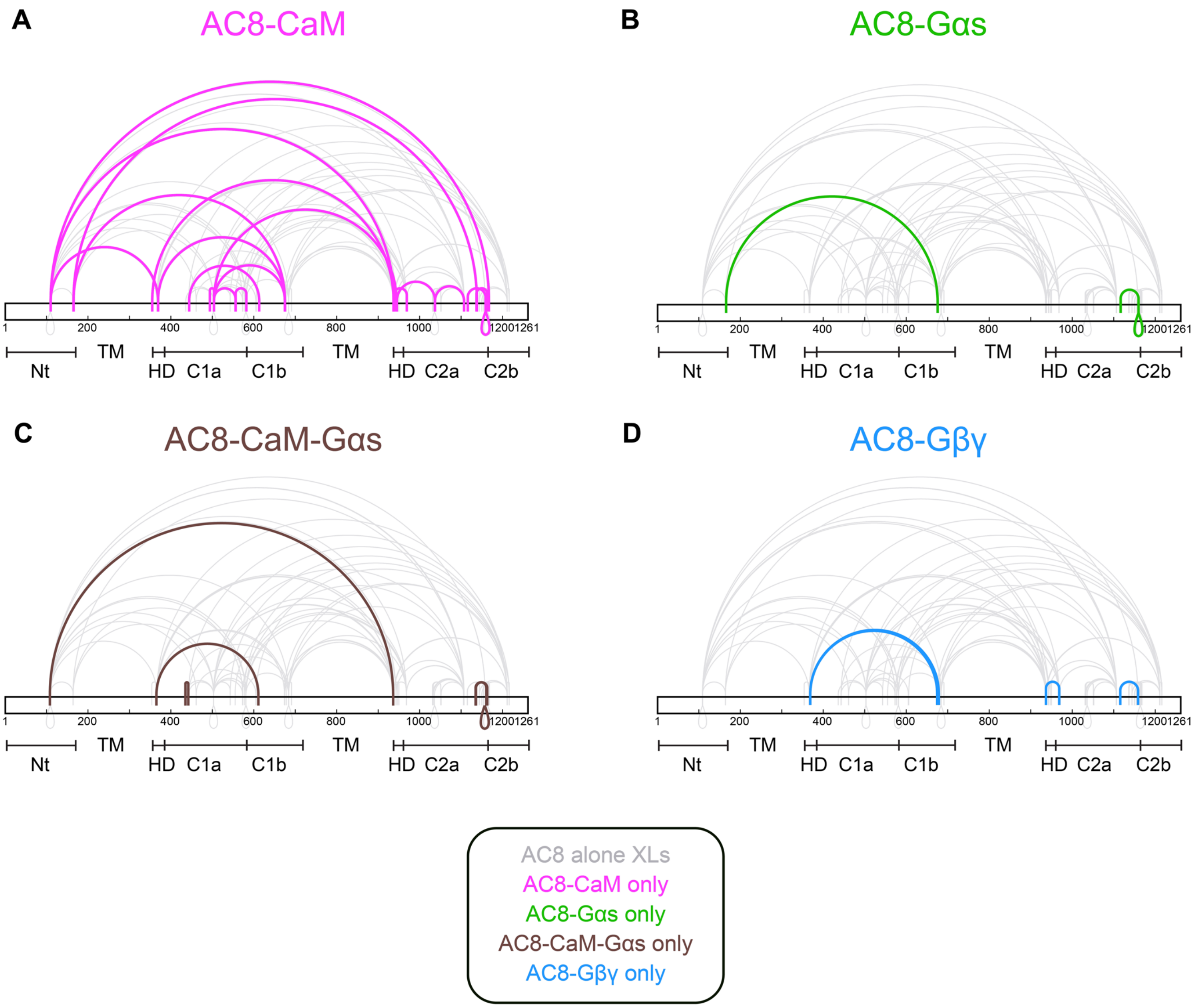

Figure EV4. XL-MS comparison of identified crosslinks between AC8 by itself and interactors.

(**A**) Crosslinks detected in the AC8 only sample (no added interactor) are coloured in light grey, positioned in the background. Crosslinks only found in the AC8-CaM sample (not in the AC8 alone sample) are coloured in pink. AC8 domains and their respective locations are indicated below. (**B**) Crosslinks only found in the AC8-Gαs sample are coloured in green. (**C**) Crosslinks identified only in the AC8-CaM-Gαs sample are highlighted in brown. (**D**) Crosslinks unique to the AC8-Gβγ sample are coloured in blue.

 © The Author(s)

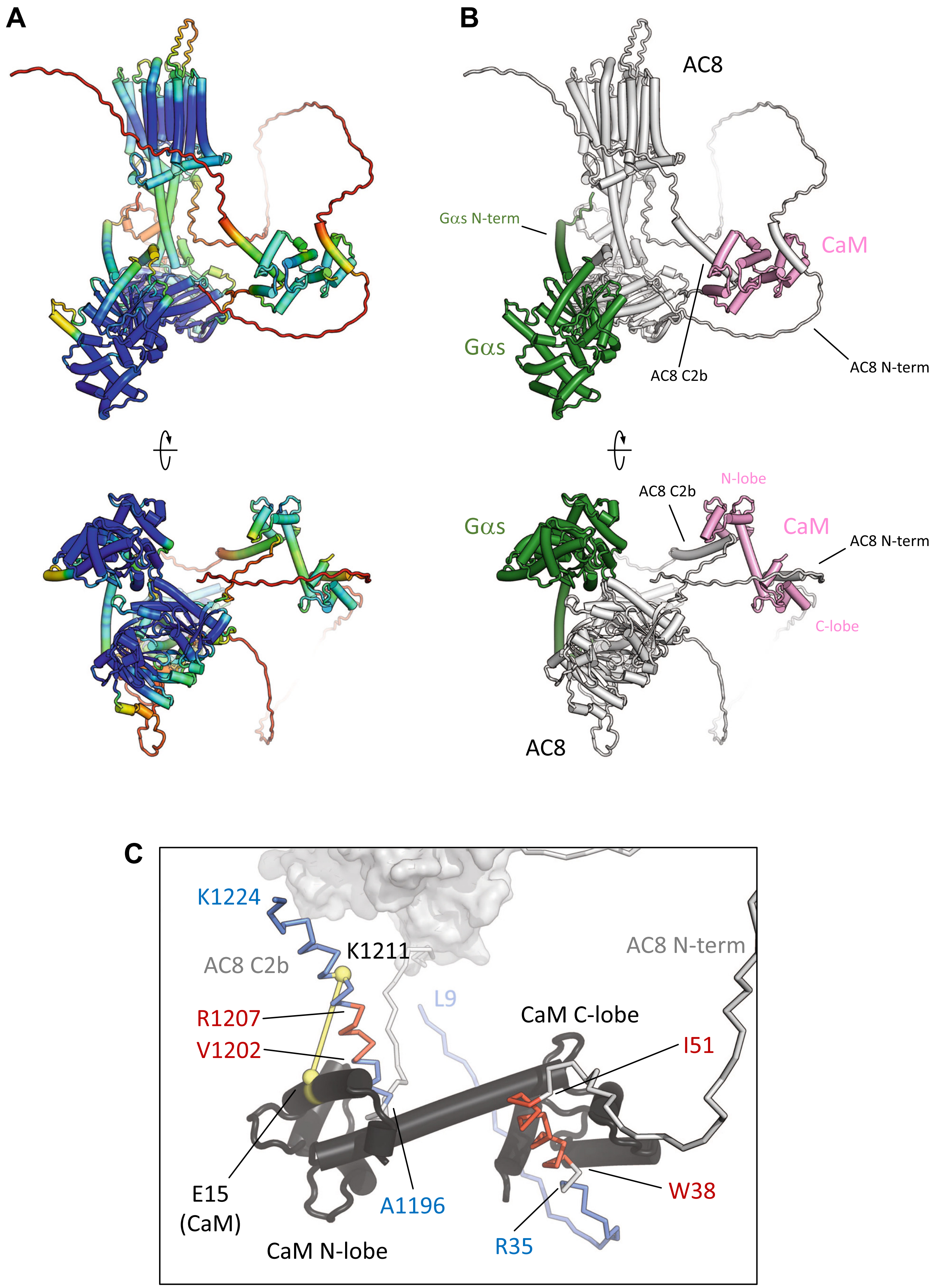
A
B
AC8
Gαs N-term
CaM
Gαs
AC8 C2b
AC8 N-term
N-lobe
AC8 C2b
Gαs
CaM
AC8 N-term
C-lobe
AC8
C
K1224
K1211
AC8 C2b
AC8 N-term
L9
R1207
CaM C-lobe
V1202
I51
W38
E15
(CaM)
A1196
R35
CaM N-lobe

Figure EV5. AlphaFold2 model of AC8-CaM-Gαs.

(**A**) The views of the model of the complex generated using AlphaFold2 as described in Materials and Methods, coloured according to the pLDDT scores (blue – high pLDDT / confidence; red – low pLDDT / confidence). (**B**) The same views as in a, with key elements of the predicted structure labelled, and individual proteins coloured white (AC8), pink (CaM) and green (Gαs). (**C**) The AlphaFold2 prediction matches well the known CaM binding sites (the 1-5-8-14 motif (WXXXVXXIXXXXXI) residues 38–51; the IQ-like motif (VQXXXR) 1202-1207, red (MacDougall et al, 2009)) and the LiP-MS-detected CaM binding peptides (9-35, 1196-1224, blue), respectively, in the N-terminus and C2b domain of AC8. CaM is coloured dark grey. The crosslink between E15 (CaM) and K1211 (AC8) detected in our XL-MS analysis is indicated as a yellow line. The distance between the Cα atoms of these residues (yellow spheres) is 17.5 Å.

 © The Author(s)
